# Dr. Mossman, on Apoplexy

**Published:** 1803-05-01

**Authors:** Geo. Mossman

**Affiliations:** Bradford


### *To the Editors of the Medical and Physical Journal.*

Gentlemen,

It was expected that the late Controversy on *Apoplexy* would have been followed by a summary comment from the pen of the Editors.* Your opinions would have had much authority; and the extended circulation of the Journal would have given them a wide publicity. The occurrence of *Apoplexy* is, perhaps, becoming more and more frequent; and from the sudden manner in which it makes its approaches, it will generally fall to the lot of the *nearest* practitioner (whatever may be his youth and inexperience) to decide on the *first* and the *most effectual means* of combating this awful disease. Much, therefore, depends on his being properly informed of the *nature* and *cure* of *Apoplexy*; for if the important stakes of life and death are to be lost or won by his *first* exertions, surely those ought to be directed by enlightened views of the subject. Now, I am persuaded, that the reader of your Journal, (and I believe that a very large proportion of professional men of the empire read the work) will be more unsettled in his faith, relative to the true nature of *Apoplexy*, and more hesitating in the application of curative agency, than he was previous to his perusal of the controversial papers which have appeared on the disease. It may be observed, that nothing so successfully eliminates the truth as this mode of agitating speculative questions; but surely, those discussions, which are so intimately blended with human happiness and human life, should be conducted with much impartiality, coolness, and candor. It is not possible, I think, to apologize for a writer, who, in a matter of so much seriousness, seeks merely to obtain a triumph, by the unjustifiable means of involving the subject in deeper mystery. If the truth is ever to be thus pitifully sacrificed, it ought to be, when the question does not so materially direct the destinies of the human species.

In the papers on *Apoplexy*, in your Journal, there is a strange diversity of opinion. Upon subjects purely specu-

* The Editors did intend to have given an opinion on the subject, but they soon found that any opinion would be unsatisfactory to many of their readers, and that some might think them prejudiced or partial. They are glad to see the subject in the hands of Dr. Mossman, but doubt whether all his readers will agree in opinion with him.

lative, when a recurrence to facts is impossible, this want of agreement may be expected; but, it is very remarkable indeed, that there should be so little unanimity about the *cause* and the *cure* of *Apoplexy;* for, the morbid phænomena of this disease, both before and after death, are so uniform, as to draw from every author, a description very exactly similar. It frequently happens that our observations, and even our experiments, are made to bend to some preconceived opinions; but the living and the dead appearances of *Apoplexy,* are, it seems, too striking, to admit of this deception; we are agreed about both. The *cause* and the *cure,* are the points to be settled; and in the prosecution of this important investigation, the mind of the enquirer should have no particular doctrine to serve, nor any particular point to carry. He should examine, arrange, and compare; and having done so, he should state facts as they are, and not as he, perhaps, may wish them to be. He should not contend for opinions, but for useful knowledge. He should not load the discussion by much extraneous matter, or with much loose theory; but should enlighten the subject by a faithful and correct history of what he has seen, and what he believes to be true. A parade of literature should never be substituted for a close analysis; nor should a concerted list of ancient or foreign authorities ever supercede the satisfactory mode of knowing the truth by evidence and induction. I do certainly respect genius and talents in whatever clime they grow, and the names of Morgagni and Spallanzani will ever command my veneration; but it is certainly true, that foreign authorities have been adduced in a late Controversy, to which much celebrity never before attached. It is also true, that, whatever may have been the reputation of some ancient authors, the splendid discoveries which have been made in *Physiology,* since their time, must necessarily lessen the value of their opinions. There is a stateliness, I think, in quoting such authorities as Riverius or Van Helmont; and there is a want of fairness in deducing from the opinions of those men, any sound practical inferences. These are not evidences to the man, who is not satisfied till he observes and thinks for himself; and who is not impatient of the labour of doing so. But, if men will have it so, and if authorities alone are to arbitrate the question, it will not be difficult to collect an immense majority of medical voices in favor of *Apoplexy* being *exclusively a brainular disease.* My justly celebrated preceptor, Dr. Cullen, has been quoted by almost every man who took a part in in the controversy. His whole history of *Apoplexy* is strikingly luminous, and accords very exactly with what I have enumerated as the general opinion; and in his treatment of the disease, if he do not positively forbid the exhibition of *emetics* (about the employment of which there has been so much controversy) he very certainly does not recommend them. A quotation, indeed, from the manuscript lectures of Dr. Cullen, admitting of the use of *emetics*, has been communicated to us anonymously; but as it comes in this shape, it ought not to have been given at all, for it is not evidence.

During the last fifteen years, I have visited a very considerable number of patients labouring under *Apoplexy*; and although I have not preserved in writing perfect histories of all the cases which have occurred, yet I have the most complete recollection, that all of them (except those which were accompanied by phænomena so vehement as to place them out of the reach of professional aid) either experienced a temporary *alleviation* of symptoms, or eventually a *cure*, from *general* or from *local bleeding*. I ought to observe that I never administered an *emetic*.—To me this instrument appears an awful one indeed.—I should tremble to employ it. I *once*, and only *once* in my life, saw an *emetic* exhibited in a case of *Apoplexy*; my subordinate situation forbade my interference. The case was a fatal one. Since the commencement of the Controversy, which for so many months occupied the pages of your Journal, I have been consulted for four cases of *Apoplexy*. Two of my patients perished; two are alive and well. The first fatal case was that of a gentleman, of about fifty years of age, who, after a light dinner, was seized with symptoms of vehement *Apoplexy*. Previous to the arrival of a respectable practitioner, an *emetic* had been administered, and had operated. *Local bleeding*, *blistering*, and *purgatives*, were then properly, I think, though fruitlessly, employed. About fifty hours from the period of attack he perished. I visited him with Mr. Maud, a few hours before the closing of the scene.

The second fatal case was a gentleman of a full habit, and about sixty years of age; who, for several years, had been subject to slight *apoplectic* symptoms, which were very generally succeeded by symptoms of *paralysis*; and towards the latter part of his life those symptoms terminated in *hemiplegia*. From the first of his being seized, I occasionally saw him; and, from time to time, I afforded him relief by *shaving his head*, by *blisters*, *local bleeding*, and

 *laxatives*;

*laxatives*; and even at the last, when he was labouring under the phænomena of a vehement *Apoplexy*, he very certainly derived some temporary relief from the application of *leeches* to his temples. Mr. Carr assisted me in the treatment of this case from the beginning.

The third case, and a successful one, was a married woman, about forty years of age, who was seized with symptoms of *Apoplexy* in the morning, as she was going into her garden. Symptoms of *hemiplegia* succeeded to this affection. I saw her with Mr. Maud; *leeches* to the temples, *blisters*, and *drastic purgatives*, were employed with manifest and immediate advantage. For several weeks, however, the phænomena of *Apoplexy* continued occasionally to recur, and were always successfully combated by a repetition of the remedies described. My patient is now in perfect health.

The last case was a gentleman, of sixty years of age, and of a habit remarkably plethoric. He dined as usual; and as he was walking in his garden, he sunk suddenly under symptoms of an apparently vehement *Apoplexy*. A surgeon was immediately sent for, who, I think, with much propriety, took from his arm a large quantity of *blood*, and *blistered* him. At the time when he was seized I was also sent for, and I arrived at the patient's abode about two hours after he fell down. The instruments, however, which Mr. Sykes had employed, had much abated the violence of the symptoms. I advised a repetition of the same remedies, should circumstances demand their administration; and, in addititition, I recommended the immediate employment of *laxatives*. The patient soon recovered, and is now in perfect health.

I cannot quit this truly interesting subject without adverting to a measure which, I am persuaded, will be found to operate most powerfully in the prevention of this awful disease. I allude to *a limitation of the use of fluids, in habits predisposed to Plethora and Apoplexy*. I am taught, by long observation and experience, to expect effects highly beneficial from the adoption of this plan. The phænomena of *plethora* and *obesity*, are (as I have constantly observed) referable, not to the taking in of *solid*, but of *liquid* nutriment. I am persuaded that few observations are more generally true than this; and it is a rich field for future enquiry. A Mr. Davidson, many years ago, called the attention of his brethren to the effects of the limitation of fluids, in cases of *pulmonary hæmorrhage*; and from the reasoning and facts which he adduced on the subject,

I think

I think it ought to have excited more general interest. At present I shall say nothing in elucidation of the doctrine; but only observe, that a few trials will soon operate to the conviction of every practitioner, not only of its strikingly beneficial but extensive influence. A very remarkable and successful application of this doctrine was lately made in counteracting a singularly strong predisposition to *Apoplexy*. The ingenious experimenter was Dr. Ryan, of Kilkenny; and, from the Eighth Volume of your Journal, I extract the following expressive quotation:

"The efficacy of this (a partial abstraction of fluid) was prominent and striking above every other remedy employed; the pulse lost of its force, of its hardness, of its frequency and fulness. So sudden and manifest was the transition from morbid to healthy action, after the adoption of this plan, that the exemption from disease, which took place, and the prolongation of life, may be in a great measure attributed to its influence."

*Bradford,*

*March* 18, 1803.

I am, &c.

GEO. MOSSMAN.

P. S. A paper on the subject of *Febris Puerperalis* will in a few days be transmitted to you. The subject is of high interest. The *proximate cause* and *successful mode* of *cure* are still *desiderata*. This *Fever* has been little treated of in your Journal; and by the exhibition of some new matter, I hope to excite a discussion respecting it.

